# A Multi-Study Model-Based Evaluation of the Sequence of Imaging and Clinical Biomarker Changes in Huntington’s Disease

**DOI:** 10.3389/fdata.2021.662200

**Published:** 2021-08-05

**Authors:** Peter A. Wijeratne, Eileanoir B. Johnson, Sarah Gregory, Nellie Georgiou-Karistianis, Jane S. Paulsen, Rachael I. Scahill, Sarah J. Tabrizi, Daniel C. Alexander

**Affiliations:** ^1^ Centre for Medical Image Computing, Department of Computer Science, University College London, London, United Kingdom; ^2^ Huntington’s Disease Research Centre, Department of Neurodegenerative Disease, University College London, Queen Square Institute of Neurology, London, United Kingdom; ^3^ Monash Institute of Cognitive and Clinical Neurosciences, School of Psychological Sciences, Faculty of Nursing, Medicine, and Health Sciences, Monash University Clayton Campus, Clayton, VIC, Australia; ^4^ Departments of Neurology and Psychiatry, Carver College of Medicine, University of Iowa, Iowa City, IA, United States

**Keywords:** huntington’s disease, biomarkers, disease progression model, multi-study investigation, clinical staging

## Abstract

Understanding the order and progression of change in biomarkers of neurodegeneration is essential to detect the effects of pharmacological interventions on these biomarkers. In Huntington’s disease (HD), motor, cognitive and MRI biomarkers are currently used in clinical trials of drug efficacy. Here for the first time we use directly compare data from three large observational studies of HD (total *N* = 532) using a probabilistic event-based model (EBM) to characterise the order in which motor, cognitive and MRI biomarkers become abnormal. We also investigate the impact of the genetic cause of HD, cytosine-adenine-guanine (CAG) repeat length, on progression through these stages. We find that EBM uncovers a broadly consistent order of events across all three studies; that EBM stage reflects clinical stage; and that EBM stage is related to age and genetic burden. Our findings indicate that measures of subcortical and white matter volume become abnormal prior to clinical and cognitive biomarkers. Importantly, CAG repeat length has a large impact on the timing of onset of each stage and progression through the stages, with a longer repeat length resulting in earlier onset and faster progression. Our results can be used to help design clinical trials of treatments for Huntington’s disease, influencing the choice of biomarkers and the recruitment of participants.

## Introduction

The development of disease modifying treatments for Huntington’s disease (HD), a fatal neurodegenerative condition, has taken remarkable steps in recent years. There are a wide range of clinical trials attempting to validate a treatment for HD currently ongoing, including trials testing antisense oligonucleotide and micro RNA therapies (Rodrigues et al., 2020). As we move towards larger Phase III clinical trials, it is imperative that both patient recruitment and endpoint selection are targeted to ensure trials have high sensitivity to detect the efficacy of pharmacological interventions. In order to tailor cohorts and clinical trial endpoints for different therapeutic targets, we require a detailed understanding of candidate biomarkers in HD.

Onset of HD symptoms typically begins in mid-life, with individual genetic burden determining a large amount of variance in the timing of disease onset (Bates et al., 2015). It is clear that imaging and fluid biomarkers are sensitive to disease-related change many years prior to symptom onset (Tabrizi et al., 2009; Byrne et al., 2018), although the exact timing and order of these changes is still being studied. Imaging biomarkers that measure atrophy in regional brain volume show some of the largest effect sizes in both pre-manifest HD (PreHD) and manifest HD compared to other biomarker candidates, particularly in subcortical structures (Tabrizi et al., 2012; Tabrizi et al., 2013). Clinical markers assessing motor symptoms and cognitive decline typically exhibit disease-related change later than imaging biomarkers, but are currently used as primary endpoints since they have a more direct relationship with the clinical benefit of a therapy. However, when moving into large phase III trials it is important to select endpoints that relate closely to the disease stage of the patients, and biomarkers that are likely to be the most sensitive to change during this time.

Disease progression models can reveal disease-related changes at the group and individual levels directly from observed data (Oxtoby and Alexander, 2017). Here we focus on the event-based model (EBM), which infers the order in which biomarkers become abnormal from cross-sectional data. We have previously applied the EBM in HD to reveal a sequence of regional brain volume changes in the TRACK-HD study, a large multi-site study of HD (Wijeratne et al., 2018). We demonstrated that three subcortical structures (the putamen, caudate and pallidum) were the first to become abnormal, followed by regions of the insula, CSF spaces, and amygdala. We have also applied the EBM to reveals the sequence of mixed biofluid, imaging and clinical changes in the HD-CSF study, a smaller single-site cohort study of HD (Byrne et al., 2018; Rodrigues et al., 2020).

However, these analyses were performed separately, and no direct comparison was made between studies to determine which features and findings were consistent. The analysis we present here is the first cross-study EBM analysis performed in HD (or any other disease), using data from the three largest imaging cohort studies in HD: TRACK-HD, PREDICT-HD and IMAGE-HD (Paulsen et al., 2008; Tabrizi et al., 2013; Poudel et al., 2015). We also add commonly used phenotypic cognitive and motor markers to the analysis to compare the stage at which these become abnormal across cohorts. Furthermore, we investigate the impact of genetic burden, as measured by cytosine-adenine-guanine (CAG) repeat length, on progression through the sequence of events. We therefore provide new information on the consistency of measurable imaging and clinical biomarker changes across differing study designs and individual-level genetic information, which has direct relevance to the design of multi-centre clinical trials in HD.

## Materials and Methods

### Cohorts

Participants from the PREDICT-HD, TRACK-HD and IMAGE-HD studies with MRI data collected at three time-points (study baseline plus two follow-ups) on the same scanner were included in the study. All scans underwent visual quality control (QC) prior to inclusion, after which there were 284 participants from four centres in TRACK-HD; 171 participants from 20 centres in PREDICT-HD; and 77 participants from one centre in IMAGE-HD. We note that no participants underwent any disease modifying treatment during data collection. Table 1 shows the demographic, clinical and cognitive data at baseline for all cohorts and groups. As noted previously (Wijeratne et al., 2020), there are differences between the groups in a number of criteria due to different recruitment strategies.

**TABLE 1:** Demographic data for the PREDICT-HD, TRACK-HD and IMAGE-HD participants at baseline. Acronyms used: HC = healthy control, PRE = preHD, HD = manifest HD, P = PREDICT, T = TRACK, I = IMAGE. TIV = Total Intracranial volume, TMS = UHDRS Total Motor Score, DCL = Diagnostic Confidence Level, TFC = UHDRS Total Functional Capacity, DBS = Disease Burden Score, SDMT = Symbol Digit Modalities Test, SWRT = Stroop Word Reading Test. A value of “-” indicates that the data were not available.

	HC_P	HC_T	HC_I	PRE_P	PRE_T	PRE_I	HD_P	HD_T	HD_I
Age	45.1 ± 10.9	46.3 ± 10.4	43.3 ± 13.6	41.8 ± 11.0	41.2 ± 8.9	39.3 ± 8.2	46.5 ± 10.7	48.5 ± 9.3	53.0 ± 7.9
Sex	25:11	58:42	17:5	85:47	55:49	15:13	3:0	43:37	7:19
TIV (l)	2.07 ± 0.2	2.12 ± 0.22	2.14 ± 0.23	2.01 ± 0.19	2.15 ± 0.22	2.05 ± 0.19	1.89 ± 0.08	2.09 ± 0.19	2.15 ± 0.28
CAG	20.44 ± 3.5	—	—	42.4 ± 2.7	43.0 ± 2.3	42.7 ± 2.0	43.3 ± 4.2	43.8 ± 3.0	42.9 ± 2.1

### TRACK-HD Study

Data for TRACK-HD were collected at four centres; Leiden, London, Paris and Vancouver between 2008–2011 (Tabrizi et al., 2013). HD gene-carriers were recruited from HD clinics and were required to have a CAG of ≥40. At baseline, 123 controls, 120 PreHD participants and 123 HD participants were recruited. PreHD participants were required to have a burden of pathology score > 250 (calculated as [age x (CAG-35.5)] (Langbehn et al., 2004), and a UHDRS Total Motor Score (UHDRS-TMS) (Huntington Study Group, 1996) of less than five, indicating minor motor symptoms. Manifest HD participants were required to have a diagnostic confidence level (DCL) of four and a Total Functional Capacity of seven or more, as measured by the UHDRS TFC (Huntington Study Group, 1996). 3T T1-weighted scans were acquired from four scanners (two Siemens, two Philips). The parameters for Siemens were TR = 2200 ms, TE = 2.2 ms FOV = 28 cm, matrix size = 256 × 256, 208. For Philips TR = 7.7 ms, TE = 3.5 ms, FOV = 24 cm, matrix size = 242 × 224, 164. The acquisition was sagittal to cover the whole-brain. There was a slice thickness of 1mm, with no gap between slices. These acquisition protocols were validated for multi-site use. The study was approved by the local ethics committees, and written informed consent was obtained from each participant.

### PREDICT-HD Study

Participants were recruited at 33 global centres, with most participants either PreHD or healthy controls (Paulsen et al., 2008). All participants were required to have had genetic testing (CAG ≥ 39 repeats) independent of the research study. PREDICT-HD recruited a total of 1,013 PreHD and 301 gene-negative controls between 2001 and 2012. Participants were excluded from the study at enrolment if there was a diagnosis of HD or evidence of an unstable illness, alcohol or drug abuse, a history of special education or central nervous system disease, a pacemaker or metallic implants, anti-psychotic medications prescribed in the previous 6 months or use of phenothiazine-derivative anti-emetic medication for 3 months or more. MRI acquisition parameters for the PREDICT-HD scanners included in this analysis are provided in (Wijeratne et al., 2020). The study was reviewed and approved by institutional review boards at all study and data processing sites. Participants underwent informed consent procedures and signed consents for both participation and to allow de-identified research data to be sent to collaborative institutions for analysis.

### IMAGE-HD Study

IMAGE-HD was a single-centre study which recruited control, PreHD and manifest HD participants (Poudel et al., 2015). Gene carriers had a CAG of ≥ 39 repeats, and PreHD and manifest HD participants were allocated to each group based on their UHDRS-TMS, with those having a score of five or less included in the PreHD group and participants with a score of greater than five included in the manifest HD group. 108 participants were recruited at baseline, with imaging data available for 31 PreHD, 31 manifest HD and 29 control participants. Data were collected using a Siemens Magnetom Tim Trio 3T scanner with a 32 channel head coil. T1-weighted images were acquired with 192 slices, 0.9 mm slice thickness, 0.8 mm × 0.8 mm in-plane resolution, TE = 2.59 ms, TR = 1900 ms, flip angle = 9°. The study was approved by the Monash University and Melbourne Health Human Research Ethics Committees and informed written consent was obtained from each participant prior to testing in accord with the Helsinki Declaration.

### Image Analysis

Structural MRI for each participant at baseline plus two follow-ups were analysed. T_1_-weighted MRI data at 3T were used from the TRACK-HD and IMAGE-HD datasets, and at 1.5T (*N* = 136) and 3T (*N* = 35) from the PREDICT-HD dataset. For each dataset longitudinal registrations were performed on each participant via SPM12 using MATLAB version 2012b. The serial longitudinal registration pipeline was applied to all participants with data from three consecutive timepoints using default settings (Ashburner and Ridgway, 2012). This registration process resulted in an average scan for each participant along with Jacobean deformation maps. For every participant, the average scan was parcellated into 156 regions using the Geodesic Information Flows (GIF) software (Cardoso et al., 2015). Each region was then multiplied by Jacobian deformation maps to create a volumetric map for every region for every time-point.

Bilateral regions were combined across hemispheres as there is little evidence of hemispheric differences in HD atrophy (Minkova et al., 2017; Minkova et al., 2018). To enable interpretation of our results, we included a subset of biomarkers in this analysis based on HD pathology. These were the putamen, caudate, pallidum, lateral ventricles and global white matter. Total intracranial volume was calculated as the sum of cerebrospinal fluid (CSF), cortical gray matter, deep gray matter, and white matter (WM). All scans, registrations and segmentations underwent visual QC to remove scans due to poor quality defacing that was conducted on the MRI scans, or failures in registration and segmentation, or due to other pathology.

### Other Variables

To facilitate further comparison among the three studies, three additional measures of phenotypic progression from the Unified Huntington’s Disease Rating Scale (UHDRS) that were available from all three cohorts were included. The UHDRS Total Motor Score (TMS) was used to measure motor symptoms (Huntington Study Group, 1996). Two cognitive scores from the UHDRS—the symbol digit modalities test (SDMT) (Smith, 1991) and stroop word reading test (SWRT) (MacLeod, 1991)—were used as cognitive outcome measures, and CAG repeat length was used to quantify approximate lifetime genetic burden.

### Covariates

All imaging and clinical variables were adjusted for covariates (age, sex, site) by regressing against the HC samples in each study separately. In addition, the imaging variables in the PREDICT-HD cohort were adjusted for field strength; the imaging variables in all studies were adjusted for total intracranial volume; and the clinical variables in all studies were adjusted for level of education.

### Event-Based Model of Disease Progression

We use the event-based model (EBM; Fonteijn et al., 2012; Young et al., 2014) to infer the sequence of imaging and clinical biomarker changes in each study cohort. The EBM defines disease progression as an ordered sequence of abnormality events, which correspond to the transition of a biomarker from a healthy to abnormal state. To infer the most likely sequence of events across the population, the EBM fits healthy and abnormal distributions for each marker separately and makes the assumption of monotonic biomarker change. This assumption is reasonable for many biomarkers in progressive diseases, and in particular the imaging and clinical markers we use in this analysis.

Here we use non-parametric kernel density estimate mixture models (Firth et al., 2020) to fit the healthy and abnormal biomarker distributions, as they are more flexible than Gaussian mixture models. We fit these models to baseline data from the TRACK-HD cohort, as it provides the best sampling of HC (i.e., healthy) and HD (i.e., abnormal) groups (Supplementary Figure S1 for the distributions and fits). We then use these mixture models to infer the most likely sequence, S, for each study separately using their respective baseline cohorts, and estimate the uncertainty in the sequence ordering using Markov chain Monte Carlo sampling of the model posterior. After inferring S, we can obtain a model-based disease stage by calculating the likelihood distribution over all stages for a given individual. We then take the maximum likelihood stage as the inferred individual-level stage.

### Statistical Models of Progression

To interrogate the relationship between EBM stage and genetic burden, as specified by an individual’s CAG repeat length, we build polynomial mixed effects regression models. Specifically, we regress the inferred individual-level EBM stage against age at each time-point (not just baseline) for each CAG group separately, with individual-level random intercepts. Instead of taking the maximum likelihood EBM stage, here we take the weighted average stage, as it accommodates uncertainty in the staging; as such, the stage is a continuous measure We construct both linear and quadratic mixed effects models for each CAG group, and select the model that provides the best fit as quantified by the size of the confidence intervals.

## Results

### Event Sequences Are Consistent Across Studies

**FIGURE 1:**
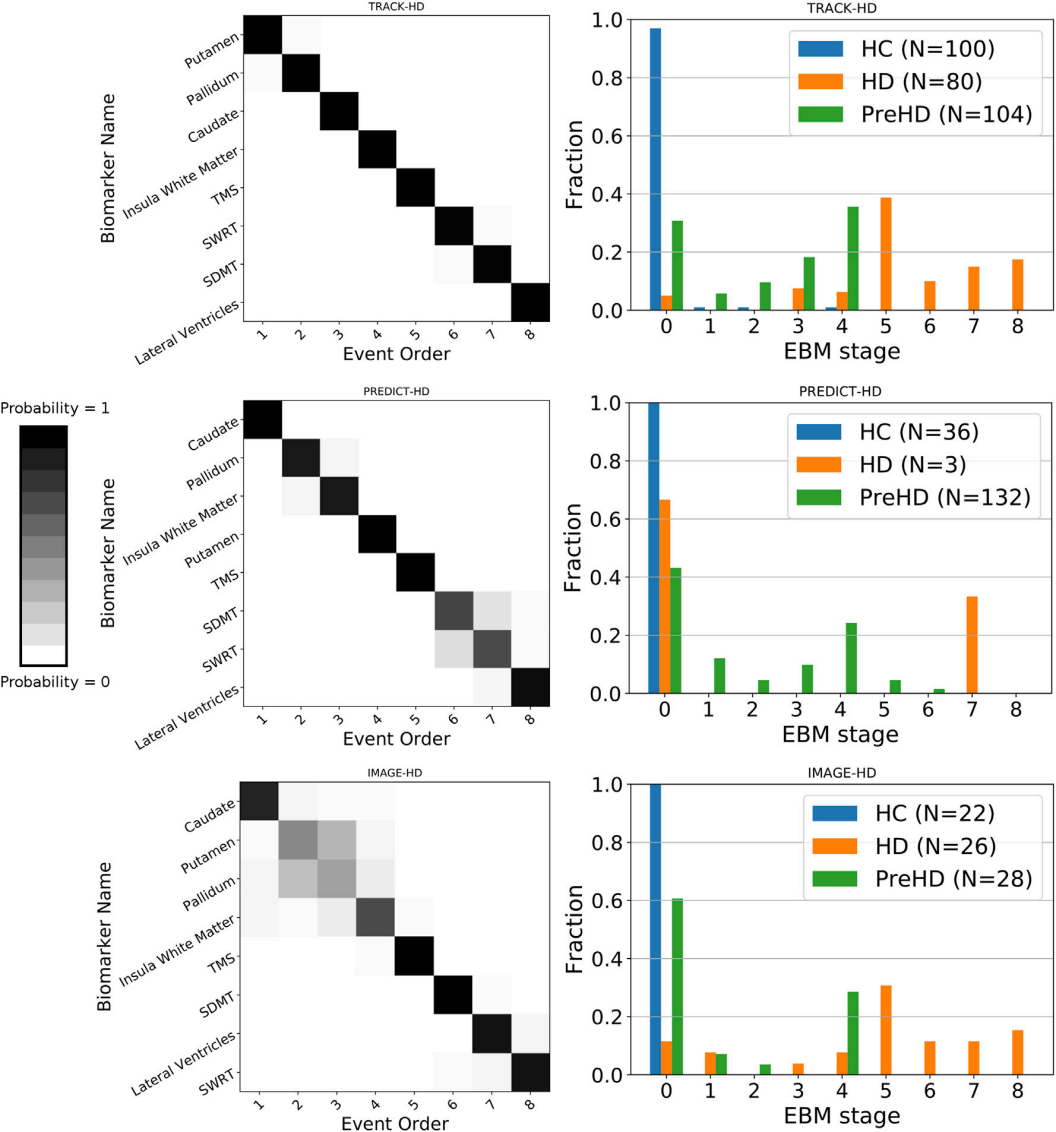
**Left column:** positional variance diagrams showing the estimated order of regional brain volume and clinical marker abnormality events in PreHD and manifest HD patients at baseline, from the TRACK-HD, PREDICT-HD and IMAGE-HD cohorts separately. The heatmaps indicate the magnitude of the probability of the ordering; dark diagonal boxes indicate strong event ordering, and lighter indicate possible event permutations with strength proportional to the off-diagonal boxes. **Right column:** individual-level disease stage for each group in each cohort, predicted by the EBM sequence fit to each cohort separately.

We find sequences of clinical and imaging events that are remarkably consistent across all three studies (Figure 1 left column). For all three studies, the imaging biomarkers were placed before the clinical biomarkers with the exception of the lateral ventricles, which were positioned either last or second last for all cohorts. TMS was the fifth marker to become abnormal for all three cohorts, with SDMT and SWRT in variable positions after TMS. To quantify the similarity between event sequences, we calculated the Kendall’s tau distance between each sequence separately, which returned values of 0.5 (TRACK-HD vs. PREDICT-HD, IMAGE-HD vs. PREDICT-HD) and 0.57 (TRACK-HD vs. IMAGE-HD), indicating positive correlations across all studies.

### Event-Based Model Stage Reflects Clinical Stage

We find that EBM successfully stages individuals according to their clinical stage (HC, PreHD, or HD) in all three studies, when taking the maximum likelihood stage for each individual (Figure 1 right column). As expected, the HC group is staged at or near zero, the PreHD group at intermediate stages, and the HD group across the later stages. The only exception is in the HD group in the PREDICT-HD cohort, where two of the three HD individuals are staged at zero; this is due to a combination of mismeasurement in the insula white matter and control-like clinical measurements for one individual, and mostly control-like volumetric and clinical measurements for the other individual.

### Event-Based Model Stage Is Related to Age and Genetic Burden

**FIGURE 2:**
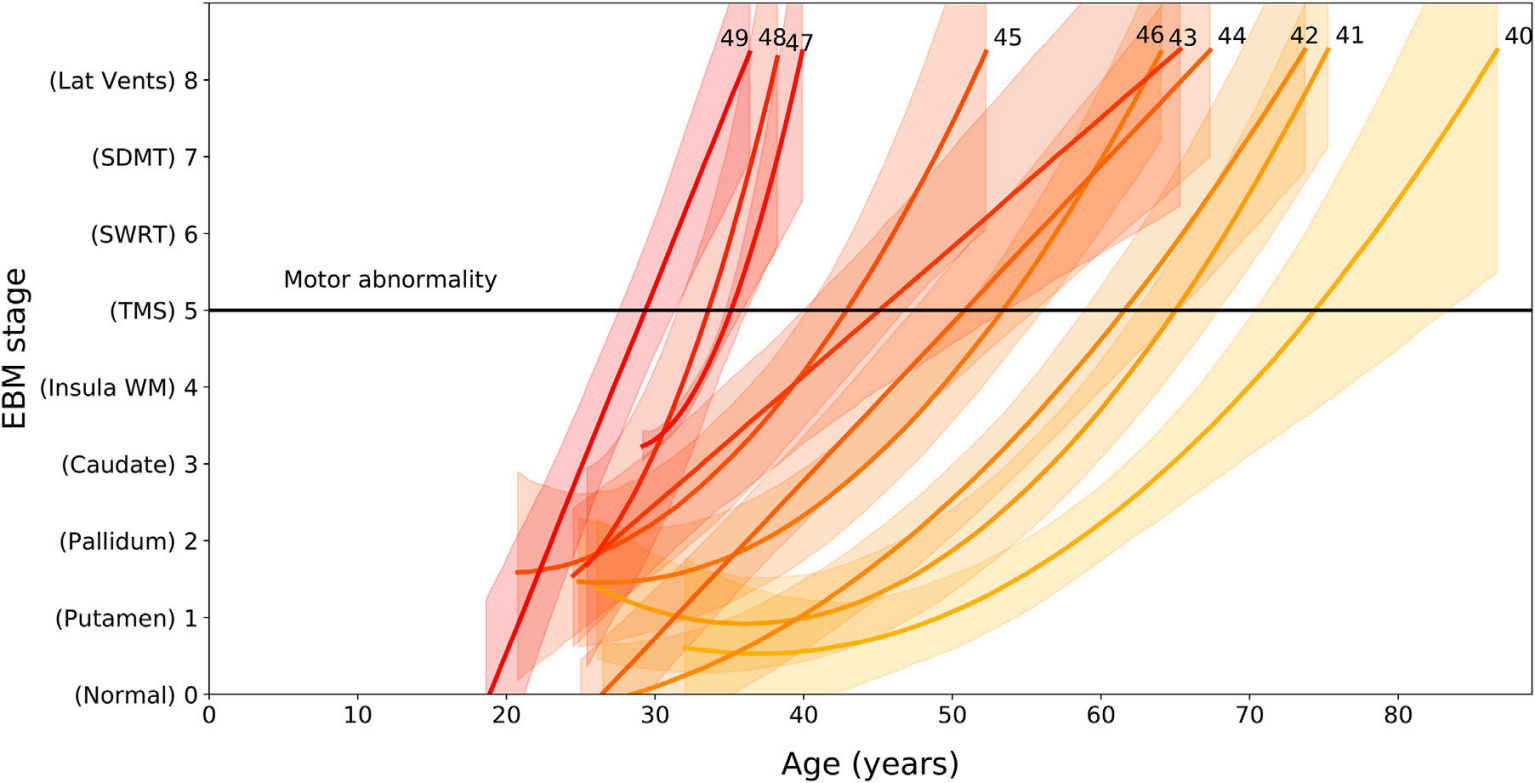
EBM stage as a function of age and CAG repeat length, for PreHD participants with at least one follow-up across all years in the PREDICT-HD, TRACK-HD and IMAGE-HD cohorts. Polynomial mixed effects models are fit to each CAG group separately, which are coloured from low CAG repeat count in light yellow to high CAG repeat count in dark red, with the CAG repeat count denoted by integer values at the end of the curves. Stages are ordered along the vertical axis according to the ordering obtained by the EBM applied to the TRACK-HD cohort (Figure 1). The stage at which TMS becomes measurably abnormal is indicated by a black horizontal line (stage 5).

We find that EBM stage and rate of progression depends on age and CAG length, with higher CAG lengths resulting in faster progression through the sequence (Figure 2). We can use the regression models shown Figure 2 to calculate the average group-level age at each event as a function of CAG repeat length. We denote the onset of motor symptoms as equivalent to the event at which TMS becomes measurably abnormal (stage 5). Note that the dependency of motor onset on CAG is not smoothly monotonic (in particular CAG = 46); this is due to small sample sizes for these CAG lengths causing variability in the regression fits.

## Discussion

Here we applied a disease progression model, the EBM, to infer the patterns of change in brain and cognitive markers across multiple cohorts in HD, and evaluated the consistency and genetic correlation of these changes. This is the first such cross-study model analysis in HD, and our findings suggest that the measurable changes in imaging and clinical volumes are largely independent of study protocols and cohort inclusion criteria. This has implications for large multi-centre clinical trials, which are necessary in HD due to its low prevalence, and suggests that the imaging and clinical biomarkers used in this analysis are suitable candidates for tracking disease progression.

Previously, we demonstrated that subcortical volumes become abnormal prior to other brain regions, which was supportive of the HD literature (Tabrizi et al., 2013; Byrne et al., 2018; Rodrigues et al., 2020). By applying the EBM to multiple cohorts we demonstrated that subcortical imaging biomarkers become abnormal prior to clinical markers. Across the three cohorts, the position of the caudate, pallidum, putamen and insula white matter varied in their position, but were consistently placed prior to clinical markers. The lateral ventricles were placed last (TRACK-HD, PREDICT-HD) or second to last (IMAGE-HD). The three non-imaging biomarkers are all ranked after the subcortical and white matter measures, with TMS first of these measures in all three cohorts. The differences in the relative positions of each imaging change across studies may be due to subtle between-sample variances related to cohort characteristics or imaging acquisitions, but by analyzing all data *via* the same imaging pipeline we can rule out the effects of different post-processing procedures. These results highlight the importance of using imaging biomarkers in clinical trials recruiting PreHD and early manifest HD participants, as clinical changes may not be sensitive enough to detect the pharmacological impacts of a therapy. Currently, the majority of clinical trials are focussed on manifest HD patients, but the end-goal of a number of therapeutic approaches is to treat PreHD individuals in order to delay or halt symptom onset. Trials for PreHD patients are unlikely to detect significant changes in clinical endpoints, and thus should also include imaging biomarkers as priority endpoints. The nature of these endpoints may vary dependent on the pharmaceutical mechanisms, but our results suggest that there are a variety of candidate regions available that change prior to clinical measures.

Importantly, we also demonstrate that the rate of progression through these stages is largely dependent on CAG repeat length, with wide variation seen in the age at which HD gene carriers with different CAG repeat lengths might be expected to pass through each stage. Our analysis of the link between CAG length, age and progression through the stages of our EBM suggest that those with shorter CAG repeat lengths undergo slower progression than those with longer CAG lengths. While this is supportive of previous work (Penney et al., 1997; Ruocco et al., 2008; Langbehn et al., 2011; Henley et al., 2012; Langbehn et al., 2019), Figure 2 demonstrates how significantly this varies. Those with a CAG repeat length of 49 are expected to have abnormal sub-cortical and WM volumes by approximately 27 ± 2 years of age, while those with a CAG repeat length of 40 are estimated to be approximately 70 ± 5 years of age at the same stage. This large variability indicates that participants with larger CAG repeat lengths are expected to show faster progression during a clinical trial, and this should be considered during recruitment and treatment evaluation.

There are limitations to the analysis we present here. Firstly, we do not include biofluid biomarkers, such as neurofilament light, since these measures are only available for a limited selection of TRACK-HD data, and not at all for PREDICT-HD and IMAGE-HD. However, in previous work we demonstrate that these markers appear to be the first to show abnormalities in HD (Byrne et al., 2018; Rodrigues et al., 2020). In addition, we limited our investigation to a subset of available imaging biomarkers. This was done to aid interpretation, but different pharmacological mechanisms may require the consideration of other biomarkers not included here. Methodologically, we applied the basic cross-sectional EBM and hence were only able to recover the order of events, but not the time between them. Future work will use the recently developed temporal EBM (Wijeratne and Alexander, 2020) to properly leverage longitudinal data, allowing the time between events to be estimated. Finally, the basic EBM only considers a single sequence across the whole sample; it would be interesting to apply the subtyping version of the EBM (SuStaIn; Young et al., 2018) to investigate the possibility of multiple within-cohort subtypes.

By applying the EBM to multiple HD cohorts, we have confirmed that imaging biomarkers become abnormal prior to clinical and cognitive markers, and that there is large variation due to CAG repeat length in the age at which these markers become abnormal. By understanding both the sequence of changes in these markers and the correlation between the predicted individual-level stage and genetic burden, biomarkers can be more effectively selected for clinical trials in HD.

## Data Availability

The datasets used in this study are available from the study authors co-ordinators (IMAGE-HD: NGK; PREDICT-HD: JSP; TRACK-HD; SJT) under a suitable data sharing agreement. Code used to run the Event-Based Model is available here: https://github.com/ucl-pond/kde_ebm.
