# Supplementary material for: A Multi-Study Model-Based Evaluation of the Sequence of Imaging and Clinical Biomarker Changes in Huntington’s Disease
Source: Front Big Data. 2021 Aug 5;4:662200. doi: 10.3389/fdata.2021.662200 (PMC8374237; doi:10.3389/fdata.2021.662200)
Supplement: Supplementary file 2 [file DataSheet2.docx]

| **Leadership and Infrastructure** | |
| --- | --- |
| **Principal Investigator** | |
| Jane S. Paulsen | University of Iowa |
|  | |
| **PREDICT Steering Committee Members** | |
|  |  |
| **2008-2014** 3^rd^ NIH grant |  |
| H. Jeremy Bockholt  Thomas Brashers-Krug  Phil Danzer  Megan Smith  Jeffrey D. Long | University of Iowa  University of Iowa  University of Iowa  University of Iowa  University of Iowa |
| Hans J. Johnson | University of Iowa |
| Amanda Miller | University of Iowa |
| Kelsey Montross | University of Iowa |
| Holly Westerveld | Brown University |
|  |  |
| **2004-2007** 2^nd^ NIH grant and CHDI |  |
| Elizabeth Aylward | University of Washington, Seattle |
| Kevin Biglan | University of Rochester |
| Robi Blumenstein | HighQ/CHDI |
| Elise Kayson | University of Rochester |
| Hans Johnson | University of Iowa |
| Bernhard Landwehrmeyer | University of Ulm, Germany |
| Blair Leavitt | UBC, Canada |
| Marcy MacDonald | MGH, Boston |
| Kevin Duff | University of Iowa |
| James Mills | University of Iowa |
| Christopher Ross | Johns Hopkins University |
| Ethan Singner | HighQ |
| Margaret Sutherland | NINDS |
| Dan Van Kammen | HighQ/CHDI |
|  |  |
| **1999-2003** 1^st^ NIH grant |  |
| Elizabeth Aylward | University of Washington, Seattle |
| Mark Guttman  Michael Hayden | University of Toronto, Canada  University of British Columbia, Canada |
| Elise Kayson | University of Rochester |
| Karl Kieburtz  Douglas Langbehn  Martha Nance | University of Rochester  University of Iowa  Hennepin Hospital, Minneapolis |
| David Oakes  Christopher Ross | University of Rochester  Johns Hopkins University |
| Aileen Shinaman | University of Rochester |
| Ira Shoulson | University of Rochester |
| Julie Stout | Indiana University, Bloomington |
|  |  |
| **2014-2017** (Leadership for Ancillary Grants) |  |
| H. Jeremy Bockholt | University of Iowa |
| Hans Johnson | University of Iowa |
| Daniel Sewell | University of Iowa |
| Ying Zhang | Indiana University |
| Roland Zschiegner | University of Iowa |
| Kelsey Montross | University of Iowa |
| Deborah Harrington | UCSD |
| Jessica Turner | Georgia State University |
|  |  |
| **Core Sections** |  |
|  |  |
| **Biomarker Working Group** |  |
| Jane Paulsen | University of Iowa |
| Stefano DiDonato | Fondazione IRCCS Institute of Neurology |
| Ken Evans | Ontario Cancer Biomarker Network |
| Daniel Chelsky | Caprion |
| Rick Meyers | Boston University |
| Dean Jones | Emory University |
| Clemens Scherzer | MGH |
| Andrew Juhl | University of Iowa |
| Beth Borowsky | CHDI |
| Blair Leavitt | Centre for Molecular Medicine/Therapeutics, UBC |
| Wayne Mattson | E N Rodgers Mem Hospital & Bedford VAMC |
| Asa Petersen | Lund University |
| Sarah Tabrizi | National Hospital for Neurology Neurosurgery UK |
| David Weir | UBC |
|  |  |
| **Brain Donation Working Group** |  |
| Jane Paulsen | University of Iowa |
| Anne Leserman | University of Iowa |
| Carol Moscowitz | Columbia University |
| Jean Paul G. Vonsattel | Columbia University |
| Stacie Vik | University of Iowa |
|  |  |
| **Cognitive Coordination Centers:** |  |
|  |  |
| Deborah Harrington | UCSD |
| Tamara Hershey | WUSL |
| Leigh Beglinger, Kevin Duff, Megan Smith | University of Iowa |
|  |  |
| Holly Westervelt | Brown University |
| Geoff Tremont | Brown University |
| Mary Gover | Brown University |
| Melanie Faust | Rhode Island Hospital |
| Greg Elias | Rhode Island Hospital |
| Jennifer Davis | Brown University |
| Susan Bonner | Rhode Island Hospital |
| Rachel Bernier | Rhode Island Hospital |
|  |  |
| Julie C. Stout | Indiana Univeristy |
| Noelle Carlozzi | Indiana Univeristy |
| Shannon A. Johnson | Indiana Univeristy |
| Scott A. Wylie | Indiana Univeristy |
| J. Colin Campbell | Indiana Univeristy |
| Eric J. Peters | Indiana Univeristy |
| Petra Theiner-Schumacher | Indiana Univeristy |
| Heather Coates | Indiana Univeristy |
| Sarah Queller | Indiana Univeristy |
| David Caughlin | Indiana Univeristy |
| Terren Green | Indiana Univeristy |
| Shelley Swain | Indiana Univeristy |
| Bethany Ward-Bluhm | Indiana Univeristy |
| Shaun Siegler | Indiana Univeristy |
|  |  |
| **Biomedical Informatics** |  |
| H. Jeremy Bockholt | University of Iowa |
| Roland Zschiegner | University of Iowa |
| Paul Allen | University of Iowa |
| Sudharshan Reddy Bommu | University of Iowa |
| Erin Carney | University of Iowa |
| Robert Connell | University of Iowa |
| Dan Kitzman | University of Iowa |
| Bill McKirgan | University of Iowa |
| Karen Pease | University of Iowa |
| Benjamin Rogers | University of Iowa |
| Adam Scott | University of Iowa |
| Mark Scully | University of Iowa |
| Jim Smth | University of Iowa |
| Austin Suiter | University of Iowa |
| Kent Williams | University of Iowa |
| Shuhua Wu | University of Iowa |
| Ryan Wyse | University of Iowa |
|  |  |
| **Ethics** |  |
| Cheryl Erwin | University of Iowa, U TX, Houston |
| Patricia Backlar | Portland State university |
| Debbie Bury | Chicago-Kent College of Law |
| Jim Calhoun | President, HDSA Georgia |
| Vicki Hunt | Wake Forest University |
| H. Jeremy Bockholt | University of Iowa |
| Thomas Brashers-Krug | University of Iowa |
| Janet K. Williams | University of Iowa |
| Martha Nance | Hennepin Hospital, Minneapolis |
| Jason Evans  Kimberly Quaid | University of Iowa  Indiana University, Indianapolis |
| Steven Hersch | Emory University |
| Lisa Hughes | University of Texas, Houston |
| Carl Leventhal | Indiana University |
| Bernard Lo | University of CA |
| Erik Parens | The Hastings Center |
| Harold Shapiro | Princeton University |
| Aileen Shinaman | University of Rochester |
| Alice Wexler | UCLA |
| Nancy Wexler | Columbia University |
| Jane Paulsen | University of Iowa |
|  |  |
| **Iowa Study Coordination Center:** |  |
| Elizabeth Penziner | University of Iowa |
| Christine Werling | University of Iowa |
| Karla Anderson | University of Iowa |
| Craig Stout | University of Iowa |
| Kristine Bjork | University of Iowa |
| LeeAnn Davis | University of Iowa |
| Ann Dudler | University of Iowa |
| Jamy Schumacher | University of Iowa |
| Steve Blanchard | University of Iowa |
| Phil Danzer | University of Iowa |
| Kelsey Montross | University of Iowa |
| Brenda Humble | University of Iowa |
| Steve Blanchard | University of Iowa |
| Bryan Ludwig | University of Iowa |
| Anne Leserman | University of Iowa |
| Lynda Sherman | University of Iowa |
| Carissa Nehl, BS | University of Iowa |
| Jane Paulsen | University of Iowa |
| Daniel Fernandez-Baca | University of Iowa |
| Gloria Ellis | University of Iowa |
| Stacie Vik | University of Iowa |
|  |  |
| **MGH DNA Lab:** |  |
| Marcy MacDonald  James Gusella | MGH, Harvard University, Boston  MGH, Harvard University, Boston |
|  |  |
| **HSG Clinical Trials Coordination Center:** |  |
| Keith Bourgeois | University of Rochester |
| Catherine Covert | University of Rochester |
| Susan Daigneault | University of Rochester |
| Karl Kieburtz | University of Rochester |
| Elise Kayson | Elise Kayson, MS, RNC |
| Elaine Julian-Baros | University of Rochester |
| Beverly Olsen | University of Rochester |
| Constance Orme | University of Rochester |
| Tori Ross | University of Rochester |
| Karen Rothenburgh | University of Rochester |
| Joseph Weber | University of Rochester |
| Hongwei Zhao | University of Rochester |
|  |  |
| **IMAGING Working Group:** |  |
| Elizabeth Aylward | University of Washington, Seattle |
| Vince Magnotta | University of Iowa |
| Hans Johnson | University of Iowa |
| Christopher Ross | Johns Hopkins University |
| Stephen Rao | Cleveland Clinic, Ohio |
| Jeremy Bockholt | University of Iowa |
| Peg Nopoulos | University of Iowa |
| Jatin Vaidya | University of Iowa |
| Deborah Harrington | UCSD |
| Andy Feigen | North Shore University |
| David Eidelberg | North Shore University |
| Vince Calhoun | MIND Research Network, UNM, GSU |
| Jessica Turner | Georgia State University |
| Michael Miller | Johns Hopkins University |
| CF Westin | Harvard Medical School |
| Guido Gerig | Utah Center for Neuroimaging / NYU |
|  |  |
| **Cognitive Working Group** |  |
| Peter Como | University of Rochester |
| Julie Stout | Indiana University |
| Leigh Beglinger | University of Iowa |
| Susan Bonner | Rhode Island Hospital |
| Noelle Carlozzi | Indiana University/ University of Michigan |
| Gabriel Castillo | UCSD |
| Jennifer Davis | Brown University |
| Michael Diaz | WUSL |
| Ian Dobbins | WUSL |
| Kevin Duff | University of Iowa/University of Utah |
| Greg Elias | Rhode Island Hospital |
| Melanie Faust | Rode Island Hospital |
| Erin Foster | WUSL |
| Carissa Gehl | VAMC Iowa City |
| Nellie Georgiou-Karistianis | University of Melbourne |
| Deborah Harrington | UCSD |
| Tamara Hershey | WUSL |
| Herwig Lange | Air-Rahazentrum |
| Kirsty Metheson | University of Aberdeen |
| Deborah Moore | WUSL |
| Jessica Morison | UCSD |
| KC Rowe | Univrsity of Iowa |
| David Moser | University of Iowa |
| Karen Siedlecki | Fordham University |
| Kate Papp | Brown University |
| Peter Snyder | Brown University |
| Jason Reed | UCSD |
| Megan Smith | University of Iowa |
| Danielle Theriault | University of Iowa |
| Geoff Tremont | Brown University |
| Natalie Valle Guzman | John vanGeest Center for Brain Repair |
| Carol Manning | University of Virginia |
| Randi Jones | Emory Universtiy |
| Holly Westerveld | Brown University |
|  |  |
| **Recruitment Retention Committee:** |  |
| Michelle Fox | University of California, Los Angeles |
| Elise Kayson | University of Rochester |
| Elaine Julian-Baros | University of Rochester |
| Martha Nance | Park Nicollet Clinic |
| Jane Paulsen | University of Iowa |
| Elizabeth Penziner | University of Iowa |
| Kimberly Quaid | Indiana University |
| Amanda Barnes | Johns Hopkins University |
| Greg Suter | Hereditary Neurological Disease Centre |
| Andrea Zanko | University of California San Francisco |
| Randi Jones | Emory University |
| Melinda Kavanaugh | Washington University |
| Hillary Lipe | University of Washington |
| Terry Tempkin | University of California Davis |
| Stacy Vik, BA | University of Iowa |
| Rachel Zombor | Graylands, Selby-Lemnos, Perth |
| Sean Thompson | University of Iowa |
| Katrin Barth | University of Ulm |
| Jenny Naji | Cardiff University |
| Jane Griffith | Westmead Hospital |
| Norman Reynolds | Medical College of Wisconsin |
|  |  |
| **Statistics Working Group:** |  |
| Jeffrey Long | University of Iowa |
| Ji-In Kim | University of Iowa |
| Wenjing Lu | University of Iowa |
| James Mills | University of Iowa |
| Blair Harrison | University of Iowa |
| Spencer Lourens | University of Iowa |
| Dawei Liu | University of Iowa |
| Ying Zhang | University of Iowa |
| Kai Wang | University of Iowa |
| Douglas Langbehn | University of Iowa |
| Hongwei Zhao | Texas A&M University |
| David Oakes | Unifersity of Rochester |
|  |  |

| PREDICT-HD Investigators, Coordinators, Motor Raters, Cognitive Raters (sites ordered by sample size) |
| --- |
| **University of Iowa:** |
| Robert Rodnitzky, Henry Paulson, Ergun Uc, Justin Smock, Jessica Wood, Thomas Brashers-Krug, Jess Fiedorowicz, Eric Epping, Leigh Beglinger, Kevin Duff, Megan Smith, Jane Paulsen, Carissa Nehl Gehl, Karin Hoth, Clare Hey, Elizabeth Penziner, Beth Turner, Lynn Vining, Ania Mikos, Becky Reese, Rachel Conybeare, Sara Vander Heiden, Stacie Vik, Isabella De Soriano, Jessica Schumacher, Erica Wagner, Kimberly Bastic, Michelle Benjamin, Nancy Hale, Katie Hall, Mycah Kimble, Harisa kuburas, Jeremy Hinkel, Andrew Juhl, Stephen Cross, Angel Dominguez, Mackenzie Elbert, Terry Hayes, Jolene Luther, Amanda Miller, Pat Ryan, Emily Shaw, Kelli Thumma, Owen Wade, Courtney Hobart |
|  |
| **University of Melbourne,**  **Australia:** |
| Edmond Chiu, Phyllis Chua, Joy Preston, Anita Goh, Olga Yastrubetskaya, Samantha Loi, Stephanie Antonopoulos Andrew Gibbs, Phillip Dingjan, Kristy Draper, Chathushka Fonseka, Nellie Georgiou-Karistianis, Christel Lemmon, Liz Ronsisvalle, David Ames, John Lloyd, Angela Komiti, Andrew Gibbs |
|  |
| **University of British Columbia, Vancouver:** |
| Lynn Raymond, Joji Decolongon, Mannie Fan, Allison Coleman, Elisabeth Almqvist, Kimberley Carter, Rachelle Dar Santos, Jordana Hutchinson, David Weir, Blair Leavitt |
|  |
| **Johns Hopkins University, Baltimore, MD:** |
| Christopher Ross, Adam Rosenblatt, Lisa Gourley, Arnold Bakker, Robin Miller, Barnett Shpritz, Mark Varvaris, Abhijit Agarwal, Gregory Churchill, Maryjane Ong, Meeia Sherr, Kristine Wajda, Claire Welsh, Nadine Yoritomo, Jason Brandt, Carolin Eschenbach |
|  |
| **Hereditary Neurological Disease Centre, Wichita, KS:** |
| William M. Mallonee, Greg Suter, Judy Addison, David Palmer |
|  |
| **University of Washington & VA Puget Sound Health Care System, Seattle, WA:** |
| Ali Samii, Hillary Lipe, Rebecca Logsdon, Kurt Weaver, Thomas Bird, Rosalynn DeLeon, Emily Freney, Alma Macaraeg |
|  |
| **Emory University School of Medicine, Atlanta, Georgia** |
| Randi Jones, Joan Harrison, Stewart Factor, Cora Bush, Janet Cellar, Carol Ingram, Cathy Wood-Siverio, Timothy Greenamyre, Claudia Testa |
|  |
| **John van Geest Centre for Brain Repair, Cambridge, UK** |
| Roger Barker, Sarah Mason, Emma Smith, Natalie Valle Guzman, Gemma Cummins, Anna Di Pietro, Rachel Swain, Anna Goodman |
|  |
| **Westmead Hospital, Wentworth Ville, Sydney, Australia:** |
| Elizabeth McCusker, Bernadette Bibb, Clement Loy, Catherine Hayes, Kylie Richardson, Jane Griffith, David Gunn, Jillian McMillan |
|  |
| **University of Ulm, Germany:** |
| Bernhard Landwehrmeyer, Michael Orth, Katrin Barth, Carolin Eschenbach, Christine Held, Daniela Schwenk, Daniel Ecker, Anke Niess, Sonja Trautmann, Sigurd Suessmuth, Patrick Weydt |
|  |
| **Indiana University School of Medicine, Indianapolis:** |
| Kimberly Quaid, Melissa Wesson, Kathy Fleming, Jamalynne Stuck, Xabier Beristain, Joanne Wojcieszek |
|  |
| **Centre for Addiction and Mental Health University of Toroto, Ontario, Canada** |
| Mark Guttman, Rosa Ip, Alanna Scheinberg, Deanna Shaddick, Janice Stober, Rustom Sethna, Catherine Brown, Sheryl Elliott, Zelda Fonariov, Christine Giambattista, Albie Law, Irita Karmalkar, Sandra Russell, Joseph Sebastian, Adam Singer |
|  |
| **University of California, Los Angeles Medical Center, CA** |
| Susan Perlman, Laurie Carr, Russell Carroll, Brian Clemente, Arik Johnson, George Jackson,  Xabier Beristain |
|  |
| **University of California San Francisco:** |
| Michael Geschwind, Joel Kramer, Sharon Sha, Margaret Wetzel, Jonathan Gooblar, Mira Guzijan, Katherine Rose, Gabriela Satris, Joseph Winer, Christina Wyss-Coray |
|  |
| **National Hospital for Neurology and Neurosurgery, London, UK:** |
| Tom Warner, Stefan Kloppel, Charlotte Golding, Thomasin Andrews, Eirini Kalliolia, Elisabeth Rosser, Sarah Tabrizi, Maggie Burrows |
|  |
| **Cardiff University, Cardiff, Wales, UK:** |
| Anne Rosser, Olivia Jane Handley, Jenny Naji, Catherine Johnston, Sarah Hunt, Kathy Price |
|  |
| **University of Rochester, Rochester, New York, USA:** |
| Peter Como, Frederick Marshall, Charlyne Hickey, Amy Chesire, Carol Zimmerman, Christina Burton, Mary Wodarski, Timothy Couniham |
|  |
| **Neuroscience Unit, Graylands, Selby-Lemnos & Spec. Care Health Services, Perth, Australia** |
| Peter Panegyres, Rachel Zombor, Brenton Maxwell, Maria Tedesco, Elizabeth Vuletich, Mark Woodman, Joseph Lee |
|  |
| **Washington University – St. Louis, MO, USA** |
| Joel Perlmutter, Stacey Barton, Melinda Kavanaugh, Amy Schmidt, Shineeka Smith, Lori McGee-Minnich |
|  |
| **Clinical Genetics Centre, Aberdeen, Scotland, UK** |
| Sheila Simpsom, Zosia Miedzybrodzka, Jackie Hamilton, Mariella D'Alessandro, Lorna Downie, Gwen Keenan, Kirsty Matheson, Daniella Rae, Alexandra Ure, Vivien Vaughan |
|  |
| **University of Manchester, Manchester, UK:** |
| David Craufurd, Jenny Callaghan, Elizabeth Howard, Judith Bek, Ruth Fullam, Rhona Macleod, Andrea Sollom |
|  |
| **Columbia University Medical Center, New York, NY, USA:** |
| Pietro Mazzoni, Karen Marder, Paula Wasserman, Jennifer Williamson, Paula Leber |
|  |
| **Colorado Neurological Institute, Englewood, Colorado, USA:** |
| Rajeev Kumar, Lauren Seeberger,Carolyn Greenwald, Melissa Holtgrewe, Breanna Nickels, Jay Schneiders, Christopher O'Brien, Colleen Dingmann, Diane Erickson, Carolyn Greenwald, Deborah Judd, Terri Lee Kasunic, Kristi Malleck, Lisa Mellick, Dawn Miracle, Sherrie Montellano, Christina Reeves, ,Alan Diamond |
|  |
| **University of California – Davis, Sacramento, California, USA** |
| Vicki Wheelock, Terry Tempkin, Kathleen Baynes, Sarah Farias, Lisa Kjer, Nicole Mans, Joseph Marsano, Amanda Martin |
|  |
| **University of Alberta, Edmonton, Alberta, Canada:** |
| Wayne Martin, Sheri Foster, Marguerite Weiler, Oksana Suchowersky, Satwinder Sran, Pamela King |
|  |
| **Cleveland Clinic Foundation, Cleveland, Ohio, USA:** |
| Anwar Ahmed, Jagan Pillai, Stephen Rao, Christine Reece, Justina Baryak, Alexandra Bea, Emily Newman, Alex Bura, Michael Lengen, Lyla Mourany, Juliet Schulz, Janice Zimbelman |
|  |
| **Baylor College of Medicine, Waco, TX, USA** |
| Tetsuo Ashizawa, Joseph Jankovic, Christine Hunter, Norma Cooke, Carrie Martin, Karinna Pacheco, George Ringholtz, Cynthia Studenko, William Ondo, Nicte Mejia, Kevin Dat Nguyen-Vuong, Lynn Ratkos |
|  |
| **University of Calgary, Calgary, AB, Canada:** |
| Oksana Suchowersky, Mary Lou Klimek, Dolen Kirstein, Sharon Lockey, Sarah Furtado, Anne Louise Lafontaine, Dwight Stewart |
|  |
| **Hospital Ramon y Cajal, Madrid, Spain** |
| Justo Garcia de Yebenes, Asuncion Martinez-Descales, Marta Fatas, Christine Schwartz, Javier Alegre, Monica Bascunana Garde |
|  |
| **Hennepin County Medical Center, Minneapolis, MN, USA** |
| Martha Nance, Dawn Radtke, Deanna Norberg, David Tupper |
|  |
