# Supplementary material for: A Multi-Study Model-Based Evaluation of the Sequence of Imaging and Clinical Biomarker Changes in Huntington’s Disease
Source: Front Big Data. 2021 Aug 5;4:662200. doi: 10.3389/fdata.2021.662200 (PMC8374237; doi:10.3389/fdata.2021.662200)
Supplement: Supplementary file 3 [file DataSheet1.docx]

Supplementary Material


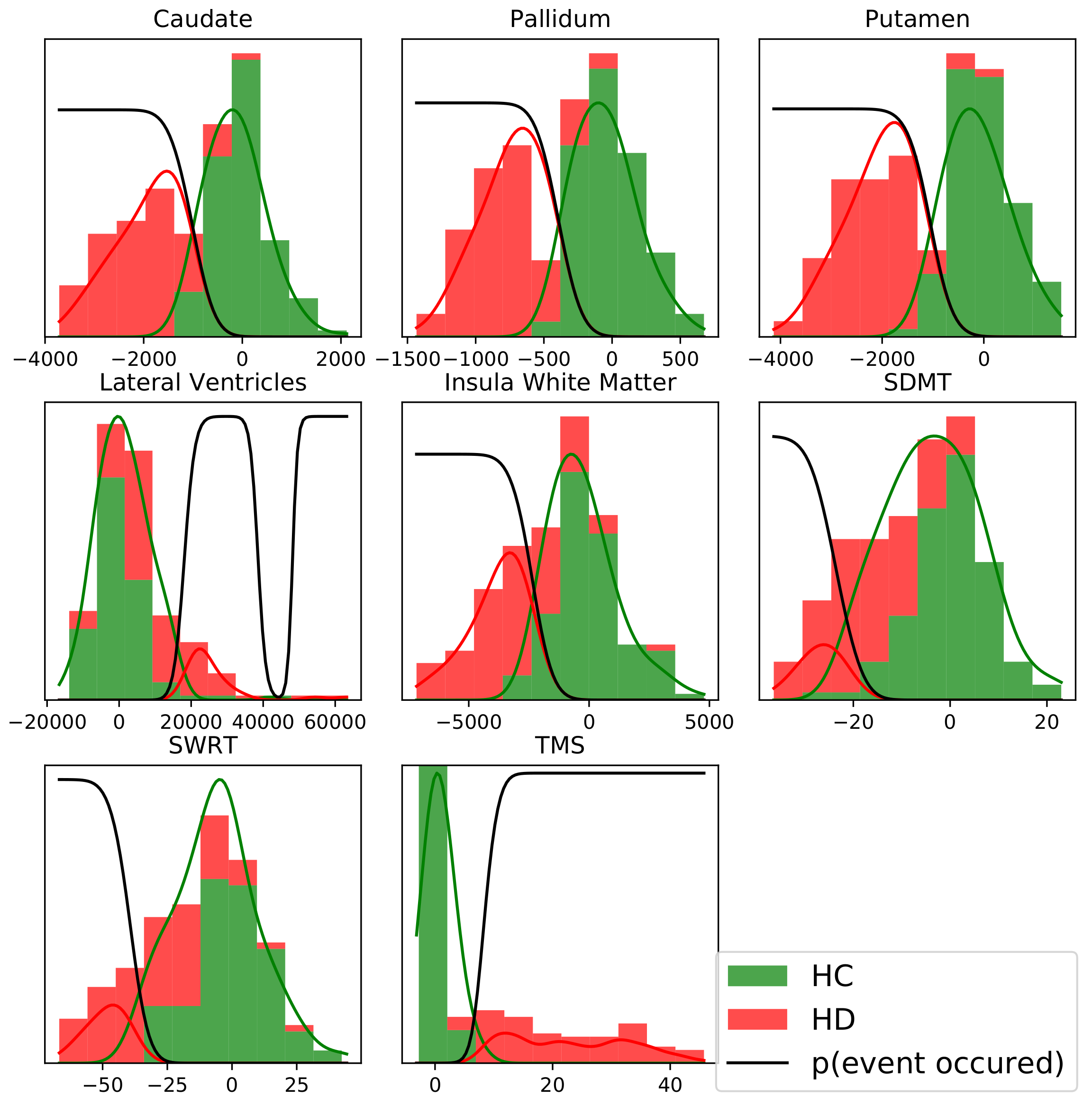


**Supplementary Figure** 1**.** Kernel density estimate (KDE) fits to the distributions of healthy control: HC (green) and manifest HD (red) groups for each biomarker, from the TRACK-HD dataset.
